# Studying the role of the Gα-protein subunit GPA-3 in amphid channel cilia of *Caenorhabditis elegans*

**DOI:** 10.1186/2046-2530-1-S1-P57

**Published:** 2012-11-16

**Authors:** A van der Vaart, S Rademakers, G Jansen

**Affiliations:** 1 Department of Cell Biology, Erasmus Medical Center, the Netherlands

## 

Many environmental cues are detected by G-protein-coupled receptors and relayed by heterotrimeric G-proteins to produce a cellular response. We recently found that a dominant active mutation in the Gα-subunit gpa-3 (gpa-3QL) affects cilia development and function. Intraflagellar transport (IFT) is required for protein transport in cilia. Two kinesin motor complexes mediate anterograde transport in cilia of *C. elegans*; kinesin-II and OSM-3 (mammalian KIF17). Together they transport particles in the middle segment of cilia, while only OSM-3 enters the distal segment where it moves at a higher speed. Several mutants have been identified, including gpa-3QL, in which the two motors move at different speeds, and thus seem uncoordinated. The IFT particle composition in the gpa-3QL mutant remains elusive and our objective is to determine this using dual-colour live imaging of fluorescently-tagged IFT proteins. Constructs have been generated to express fluorescently-tagged IFT proteins and speed measurements have shown that the dynamics of the IFT proteins are affected by their expression levels, indicating that the stoichiometry of the IFT proteins is important for their coordination. We are currently determining the optimal conditions for dual-colour live imaging in cilia of wild type animals. In addition, we have performed a suppressor screen to identify novel proteins that play a role in cilia function. We have identified the E2 ubiquitin-conjugating enzyme variant UEV-3 as a suppressor of gpa-3QL. Dye filling and cilia length are restored in the gpa-3QL uev-3 mutant. Future experiments are directed to determine the precise role of UEV-3 in cilia formation and IFT.

